# Implementation of the Texas Community-Engaged Statewide Consortium for the Prevention of COVID-19

**DOI:** 10.3390/ijerph192114046

**Published:** 2022-10-28

**Authors:** Erika L. Thompson, Bettina M. Beech, Robert L. Ferrer, Lorna H. McNeil, Jasmine J. Opusunju, Rebecca A. Seguin-Fowler, Emily E. Spence, Luis Torres-Hostos, Christopher I. Amos, Palak Desai, Jamboor K. Vishwanatha

**Affiliations:** 1 Department of Biostatistics & Epidemiology, School of Public Health, University of North Texas Health Science Center, 3500 Camp Bowie Blvd, Fort Worth, TX 76107, USA; 2 Department of Health Systems and Populations Health Sciences, University of Houston, Houston, TX 77204, USA; 3 Department of Family and Community Medicine, Long School of Medicine, University of Texas Health San Antonio, San Antonio, TX 78229, USA; 4 Department of Health Disparities Research, Division of Cancer Prevention and Population Sciences, University of Texas MD Anderson Cancer Center, Houston, TX 77030, USA; 5 CAN DO Houston, Houston, TX 77012, USA; 6 Institute for Advancing Health through Agriculture, Texas A&M AgriLife, College Station, TX 77845, USA; 7 School of Public Health, University of North Texas Health Science Center, Fort Worth, TX 76107, USA; 8 School of Social Work, University of Texas Rio Grande Valley, Edinburg, TX 78539, USA; 9 Institute of Clinical and Translational Medicine, Department of Medicine, Baylor College of Medicine, Houston, TX 77030, USA; 10 Institute for Health Disparities, University of North Texas Health Science Center, Fort Worth, TX 76107, USA

**Keywords:** COVID-19, consortium, vaccination

## Abstract

The Community Engagement Alliance (CEAL) Against COVID-19 Disparities aims to conduct community-engaged research and outreach. This paper describes the Texas CEAL Consortium’s activities in the first year and evaluates progress. The Texas CEAL Consortium comprised seven projects. To evaluate the Texas CEAL Consortium’s progress, we used components of the RE-AIM Framework. Evaluation included estimating the number of people reached for data collection and education activities (reach), individual project goals and progress (effectiveness), partnerships established and partner engagement (adoption), and outreach and education activities (implementation). During the one-year period, focus groups were conducted with 172 people and surveys with 2107 people across Texas. Partners represented various types of organizations, including 11 non-profit organizations, 4 academic institutions, 3 civic groups, 3 government agencies, 2 grassroots organizations, 2 faith-based organizations, 1 clinic, and 4 that were of other types. The main facets of implementation consisted of education activities and the development of trainings. Key recommendations for future consortiums relate to funding and research logistics and the value of strong community partnerships. The lessons learned in this first year of rapid deployment inform ongoing work by the Texas CEAL Consortium and future community-engaged projects.

## 1. Introduction 

As the COVID-19 pandemic ravaged the world in 2020, the National Institutes of Health (NIH) developed an initiative to combat health disparities. In September 2020, the NIH launched the Community Engagement Alliance (CEAL) Against COVID-19 Disparities with the mission to “(1) conduct urgent community-engaged research and outreach focused on COVID-19 awareness and education to address the widespread misinformation about COVID-19 and promote an evidence-based response to the disease, and (2) promote and facilitate the inclusion of diverse racial and ethnic populations in clinical trials, reflective of the populations disproportionately affected by the pandemic” [1,2]. This rapid response to COVID-19 health disparities was needed, as early national pandemic data showed a disproportionate burden of COVID-19 impact on racial/ethnic minorities, such as African Americans [3,4]. Moreover, historic scientific atrocities have contributed to rightful distrust of government-based scientific endeavors [3,5], such as the COVID-19 clinical trials for the vaccine. As such, the NIH initially funded 11 states in the United States to form CEAL teams to address these two overarching goals using community engagement strategies, including Texas. 

**Figure 1:**
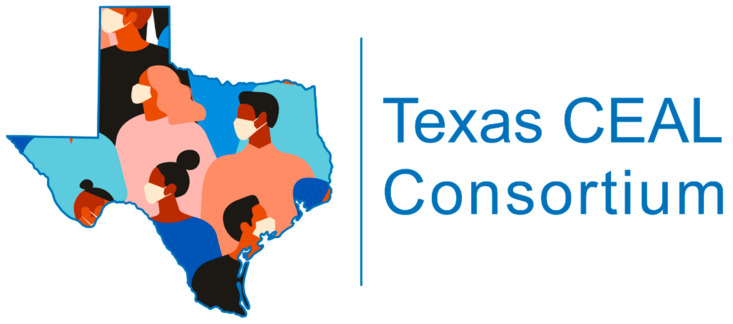
Texas CEAL Consortium.

The Texas CEAL Consortium formed in September 2020 (Figure 1). Seven projects were initially recruited from five Texas counties that had the highest morbidity and mortality for COVID-19 at the time of the project launch [6], including Bexar, Dallas, Harris, Hidalgo, and Tarrant. Projects assembled teams that had strong connections to community partners across the state so that they could rapidly engage community and assess community needs regarding COVID-19 prevention. The Texas CEAL Consortium also had an Administrative Core to liaise between the NIH and the Consortium and a Data Management and Coordination Core to provide technical assistance across the seven projects [7].

At the start of the Texas CEAL Consortium, COVID-19 vaccine trials were actively recruiting; there were no approved COVID-19 vaccines, and COVID-19 treatment strategies were continuing to be developed. Moreover, the COVID-19 pandemic has continually been plagued by misinformation on COVID-19 prevention and treatment, and widely politicized. During the first year of the Texas CEAL Consortium, the team responded and adapted to shifting priorities as COVID-19 vaccines became available, changing guidance on testing and masking, and the unpredictability of public discourse. The purpose of this paper is to describe the Texas CEAL Consortium’s activities in the first year of launching and evaluate progress to date. We also reflect on insights that may apply to future statewide initiatives to respond to public health emergencies or challenges. 

## 2. Materials and Methods

### 2.1. Texas CEAL Consortium Overview

The Texas CEAL Consortium comprised seven project teams, an Administrative Core, and a Data Management and Coordination Core. Each of the seven projects had their own individual objectives, project partners, data collection methods, and engagement and outreach activities planned (Table 1). The rationale for having separate project approaches in each county was that local researchers already had established partnerships and the community priorities may vary for each locality and population. Each project established monthly milestones that aligned with the overarching CEAL goals. The Texas CEAL Consortium identified representatives from the team to serve on National CEAL Workgroups, including Steering Committee, Assessment & Evaluation Workgroup, Communications Workgroup, and Inclusive Participation Workgroup. Ad hoc workgroups were also formed, such as the Asian American Workgroup. The Texas CEAL Consortium met bi-weekly to disseminate information across all project teams. 

### 2.2. Data Collection

Because the Texas CEAL Consortium is part of the broader CEAL initiative, frequent reporting was required. Weekly (and eventually bi-weekly) reports collected information on educational outreach activities (number of publications/posts/events and number of people reached), development of new partnerships, key accomplishments, metrics on data collection, emergent themes from data collection, and project priorities. Additionally, monthly reports reflected on activities to achieve the two CEAL goals, project successes, facilitators, barriers, and challenges. The monthly reports also collected data on the achievement of projects’ monthly milestones and whether progress had been made. Projects also reported on the partnerships they had established and maintained. All these collected data were deemed non-human subject research for the purposes of evaluation and project management.

In addition to the overall Consortium process and milestone data collection, individual projects collected research-related data relevant to their community needs [7]. Due to the collaborative nature with the national CEAL program, projects that collected survey data utilized a Common Survey instrument that included data elements related to demographics, COVID-19 prevention practices, and clinical trial participation. 

### 2.3. Evaluation

To evaluate the Texas CEAL Consortium’s progress in the first year, we utilize components of the RE-AIM Framework. The RE-AIM Framework is a useful planning and evaluation framework that can be used for a variety of health issues and programs [8]. For Texas CEAL reach, we estimate the number of people reached for data collection and education activities. Where available, we provide the racial/ethnic breakdown for the collected data. The effectiveness of the related information was determined by each project. We reflect briefly on the individual project goals and progress made in a 1-year period. For Texas CEAL adoption, we describe the partnerships established as part of the Consortium and partner engagement. For Texas CEAL implementation, we describe the outreach and education activities, including the number of products/events/posts, and engagement with our Texas CEAL website. Finally, using data on the barriers and facilitators from the monthly reports informs the implementation lessons learned on this project. 

## 3. Results

We first evaluated the *reach* of the Texas CEAL Consortium. One mode of reaching community members was through community-based data collection activities. During the one-year period, focus groups were conducted with 172 people (Table 2). Participant groups included African American and Hispanic community members, community leaders, and community health workers. Key themes included the importance of vaccine influences in the community, the power of misinformation and disinformation in the pandemic, vaccines as a communitarian act, and local organizations and trusted medical providers as being key for disseminating COVID-19 information.

Additionally, four projects used the Common Survey instrument, reaching a total of 2107 people across Texas. Nearly half of the respondents across the studies were Hispanic. Data were collected at separate time points across study sites and reflected evolving COVID-19 vaccination attitudes and uptake. 

Each of the seven projects had individualized program plans for reaching their local communities, disseminating evidence-based information, and promoting vaccine uptake. As such, each program measured their level of *effectiveness* within the respective communities. Table 3 describes each of the proposed project plans when the project launched and how the projects reported their success after Year 1. 

*Adoption* represented the partners joining the Texas CEAL Consortium. A key feature of the CEAL project was the existing and developed community partnerships during the project period. Table 1 displays the partners that were part of the Consortium. Partners represented various types of organizations (Table 2), including 11 non-profit organizations, 4 academic institutions (including higher education and public schools), 3 civic groups, 3 government agencies (including health departments), 2 grassroots organizations, 2 faith-based organizations, 1 clinic, and 4 organizations that were categorized as other. We asked the seven projects to rate the level of engagement with each of their partners. Among 22 of the partners assessed, 36% had a *coordination* level of engagement (share information and resources, frequent communication, some shared decision making), and 32% had a *coalition* level of engagement (share ideas and resources, frequent and prioritized communications, all members had vote in decision making); a total of 23% had a *collaboration* level of engagement (frequent communication was characterized by mutual trust, and consensus was reached on all decisions), and 4% were at a *networking* level of engagement (loosely defined roles, decisions made independently). 

The main facets of *implementation* consisted of education activities for local communities related to COVID-19 prevention, vaccination, and clinical trials. The Texas CEAL Consortium utilized a range of outreach modalities, including social media posts, community events, traditional media, and community trainings (Table 2). For social media, several of our local CEAL teams developed their own social media accounts that were tailored to community messaging, such as Unidos Contra COVID-19 and Operation COVID-19 Shield. Additionally, CEAL teams relied on existing and new community partnerships to host or provide resources as community events, especially COVID-19 vaccine and registration events in communities of need. Projects also held webinars and other town halls, which provided evidence-based information across the state. For example, the Texas CEAL Consortium held a town-hall style webinar with diverse presenters, including a congressperson, religious leader, community health worker, NIH official, and researcher, that reached over 500 people.

Moreover, the projects were active in local and state-level traditional media, including newspapers and television interviews, which could reach a broad spectrum of the population. For example, one project feature was the Texas COVID-19 Information and Resource Hub website (texasceal.org), which included a web-based repository (database) of COVID-19 products with attention focused on serving groups disproportionately affected by COVID-19-related health disparities. A diverse, multisector community advisory board (CAB) provided critical guidance in the development/refinement of the website, including feedback and recommendations to improve its architecture, content, functionality, and user experience. Website development also included document analyses (i.e., rubric tool) to assess the quality and cultural appropriateness of the website’s repository materials. Moreover, projects collaborated with community health workers (CHWs) for the delivery of evidence-based information to the local community. In Houston, CHWs not only provided up-to-date information on COVID-19 prevention but also connected community members to wrap-around services that supported basic needs. This group also created a repository of COVID-19 resources that were updated continuously to meet community needs. In San Antonio, CHWs participated in COVID-19 trainings and engaged in community outreach at COVID-19 vaccine events. 

Finally, some of the projects developed specific trainings related to COVID-19. Many of the trainings were geared toward community health workers, including clinical trial navigation, vaccines 101, and basic education. The Hidalgo team developed and implemented COVID-19 Engagement and Prevention Counseling to provide evidence-based information on COVID-19 prevention, vaccination, and clinical trials. This counseling reached 471 people in Hidalgo County as of October 2021. 

Each month, projects reflected on the facilitators for and barriers to project implementation. The most common facilitators were the community engagement (n = 38), partnerships (n = 18), and collaboration (n = 13) that the teams had. For example, working with community advisory boards or other community partners was reported as key to project success. The most common barriers encountered were related to the availability of funds (n = 22), delays with institutional review boards (IRBs) (n = 16), and logistics (n = 12). Delays in funds were related to the multiple flow-throughs of funding from the federal government to individual projects and the administrative hurdles involved. Logistical barriers were also largely due to the funding process, and community organizations often had challenges with not having funds up front. As for the institutional review board barrier, the prime example was finding an IRB to cover a community organization. Typically, IRBs are housed within academic institutions; however, constraints from the academic IRBs connected with the Texas CEAL Consortium prevented coverage to CAN DO Houston. Thus, CAN DO Houston had to rely on a commercial IRB.

## 4. Discussion

This paper describes the evaluation of the Texas CEAL Consortium, which was one of the 11 states originally funded by the National Institutes of Health for community engagement and community-engaged research in response to the COVID-19 pandemic. With the partnership of seven sites across Texas and their corresponding partners, the Texas CEAL Consortium collected qualitative and quantitative data to assess community needs, disseminated evidence-based information on COVID-19 vaccination, promoted COVID-19 vaccination and clinical trials, and addressed local needs. The wide cross-section of partners and the rapid deployment of engagement activities to local communities supported consortium efforts.

This evaluation elucidated key insights as the consortium was rapidly developed with diverse partners at community, academic, and governmental levels. First, given the size of Texas, our projects needed to tailor the engagement plans to local community needs recognizing that not all communities are alike. Some of the projects relied on Community Advisory Boards or key partner agencies to inform the local work and strategies. Having a state-wide administrative core permitted coordination across the different projects and enabled locally informed engagement. Second, partners embedded in the community are absolutely essential to community-engaged research. The Texas CEAL Consortium had a variety of partner types that could meet different needs of the community and were considered trustworthy by the populations served. Community partners are eager to collaborate to share scientific data with communities, but these partnerships must be protected and valued. A key group of partners were community health workers, who were active in local communities and ready to disseminate evidence-based information. 

The third key finding was related to barriers encountered along the way. Academic and governmental bureaucracy can make it challenging to conduct rapid, community-engaged research activities. As highlighted in the evaluation, logistical barriers with institutional review board and funding delays inhibited the rapid implementation of COVID-19 community-engaged research. Similar difficulties were encountered with the California CEAL [9]. Without overcoming these barriers, researchers have difficulty “moving at the speed of trust” with community members who require financial support to rapidly respond to community needs. Another key insight during this year 1 of the consortium was that while addressing misinformation and disinformation about COVID-19 was a major priority, wrap-around and support services were just as critical, as people faced social and economic instability during the pandemic. If we do not meet our communities where they are at, we cannot provide the prevention services we strive to deliver.

An additional key finding was the need to develop strategies for establishing relationships with clinical trial sites. For many of the Texas CEAL projects, this was a new relationship, which made referrals to trials difficult. The Texas CEAL did collaborate with Baylor College of Medicine for clinical trial referrals; however, these trials were making their targets quickly and did not need additional recruitment at the time the CEAL was launched. Texas CEAL was helpful for the promotion of other population-based studies of COVID-19, such as COVPN-5002, by providing educational materials and feedback on community engagement.

It is also critical to highlight that while the Texas CEAL Consortium was rapidly convened in September 2020 with an emphasis on clinical trial enrollment, the Consortium needed to remain nimble to shifting priorities, such as the emergency approval of vaccines in December 2020 [10]. Similarly, the Consortium needed to be responsive to the changing messaging surrounding testing, variants, masking, and vaccines. These quick shifts highlighted the immense value of the Texas CEAL Consortium’s community and academic partnerships. Having strong partnerships built on a foundation of trust allowed the dissemination of new information and changes in strategies to be conducted [11,12]. For example, community health workers are trusted information channels embedded in communities. Similar findings regarding community partnerships were observed by the Colorado CEAL team, further emphasizing the importance of fostering, maintaining, and sustaining community partnerships [13]. Given that improved knowledge about COVID-19 is associated with adherence to COVID-19 mitigation strategies [14], CHWs are critical to this information dissemination. Academic institutions should consider developing alternative funding sources to maintain these relationships and investments in communities in-between project-specific funding periods. Without strong and dedicated community partners, the Texas CEAL and the CEAL Consortium would not have been able to have the reach they had. Because of these community partnerships, the Texas CEAL Consortium was able to reach a diverse spectrum of communities within Texas, including Latinx/Hispanic and African American/Black communities.

This paper should be considered in the context of its limitations. First, given the diverse nature of the Texas CEAL projects and transformation over time, we were unable to obtain overall metrics of effectiveness. This was in part due to balancing the priorities of community engagement, and information and resource dissemination compared to community-engaged research. As a result, we could not describe if activities from the Texas CEAL Consortium had a direct impact on COVID-19 prevention and vaccination activities. To overcome this issue in the future, identifying comparison counties that did not have Consortium activities could be a strategy to evaluate effectiveness. Moreover, collecting survey data with common data elements throughout all communities at the same time points could permit a stronger evaluation of processes and outcomes. Additionally, the response rate to our partnership survey was lower than anticipated. An additional limitation was represented by the shifts in metrics used over time. To reduce the burden on community and academic partners, we prioritized metrics set by funders. 

## 5. Conclusions

In summary, this paper describes the development and year-one progress of a new Texas CEAL Consortium. The insights learned in this first year of rapid deployment inform ongoing work by the Texas CEAL Consortium and future community-engaged projects. Specifically, we have several recommendations for moving forward: (1) a hub-and-spoke model for consortiums would permit coordination and local tailoring; (2) community partnerships are essential, and there is a need to listen to community voices; and (3) it is necessary to anticipate challenges with academic–governmental bureaucracy in the project timeline and develop strategies to prioritize resources for community organizations.

## Figures and Tables

**Table 1:** Description of Texas CEAL Consortium projects.

Project #	County	Partners	Data Collection Methods	Engagement and Outreach Activities
1	Harris	University of Houston, Association for the Advancement of Mexican Americans, Change Happens	Virtual dialog sessions	Webinars Educational sessions with CHWs and community partners Vaccine delivery events Vaccine information cards
2	Bexar	University of Texas San Antonio, South Central Area Health Education Center, Ella Austin Community Center, WestCare Behavioral Health, San Martin de Porres Parish, Alamo Colleges System, LEAP Internet Media Group	Survey time point 1 Survey time point 2 Focus groups	Local presentations with CHWs and community Developing FAQs for CHWs Vaccine delivery events Social media—Unidos Contra COVID-19 Newsletters and videos Community advisory board
3	Tarrant	University of North Texas Health Science Center, DFW CHW Association, YMCA of Fort Worth, Fort Worth Barber Shop, United Way of Tarrant County, Tarrant County Public Health	Survey time point 1 Survey time point 2 Focus groups	Local media Vaccine registration events Social media Health events CHW outreach Direct-mail infographics Community advisory board
4	Hidalgo	University of Texas Rio Grande Valley, Operation COVID Shield/HERS (Healthcare, Education, Research, and Services) LLC	Longitudinal surveys	Social media COVID-19 engagement and prevention counseling
5	Dallas	Texas A&M University AgriLife Extension, Dia de la Mujer Latina, University of Texas Southwestern Medical Center	Focus groups Pre–post tests	CHW training Website with resource hub Community advisory board
6	Harris	MD Anderson Cancer Center, Brentwood Baptist Church, Greater Houston AHEC, Local Community Health Worker, Texas Southern University, Black Nurses Association of Greater Houston, Harris County Precinct 2, Crestmont Park Civic Club, Links Houston Chapter, Hypothesis Haven LLC	Survey (Not Common Survey)	Clinical trial navigator COVID-19 message development Email outreach Webinar series
7	Harris	CAN DO Houston, Grow Unity Resources for Living, Dia de la Mujer Latina, Rice University, Apartment Complex Managers, University of California Merced, Baylor College of Medicine, Harris County Public Health, Houston Health Department	Survey	CHW outreach calls Wrap-around resource guide COVID-19 health education repository COVID-19 testing and vaccine events

**Table 2:** Reach, adoption, and implementation for Texas CEAL.

Measures	Metrics
***Reach***	
*Participants in Data Collection Activities*	
Focus groups	172
Common Surveys	2107
Other surveys	1814
*People Reached with Educational Activities and Outreach*	
Counseling intervention (Project 4)	471
Clinical trial training of health workers (e.g., CHWs) (Project 5)	800
Other trainings (vaccines 101, adult basic education, clinical trials)	161
Website views (as of 15 November 2021)	1190 total users 4617 page/screen views 250 cites/regions 24 countries
***Adoption***	
*Partnerships Established*	
Non-profit organizations	11
Grassroots	2
Academic	4
Government	3
Clinic	1
Faith based	2
Civic groups	3
Other	4
***Implementation***	
*Education Activities*	
Social media posts + traditional media *	703
Events	87

* Combined category due to reporting structure with CEAL Leadership.

**Table 3:** Texas CEAL program plan and goals achieved.

	Project Plan	Project Success
**1**	(A) Conduct virtual dialog sessions (B) Understand barriers and facilitators to vaccine uptake and preferences for home testing kits	(A) Completed all planned sessions and elicited key themes (B) Identified barriers and facilitators to vaccine uptake in local community and perceptions of COVID-19 home testing kits
**2**	(A) Working with community health worker groups (B) Ways to show success with different CHWs	(A) Collaborated with CHW Coalition and held vaccine events (B) Provided information to Community Response Equity Coalition, group of CHWs, and supported CHW outreach events
**3**	(A) Increase vaccine uptake in 12 zip codes (B) Education outreach via lay health workers, CHWs, and social media (C) CHW training	(A) Registered people for vaccine events (B) Education distributed through these channels (C) Weekly CBPR team reviewed and disseminated new information
**4**	(A) Implementing COVID-19 Prevention and Counseling Intervention (B) Distributing the Common Survey	(A) Implemented COVID-19 Prevention and Counseling Intervention with 471 people in Hidalgo County (B) Distributed the common survey to 233 participants
**5**	(A) Website that is culturally appropriate (B) Enroll CHWs in Clinical Trial Community Navigation training	(A) Website informed by community advisory board (B) Spanish and English language training disseminated through Texas Department of Health and Human Services reaching target enrollment of 800 people
**6**	(A) Development and testing of messages (B) Connecting clinical trial navigators	(A) Developed and tested messages, and created a messaging toolkit on COVID-19 vaccination (B) Recruited a clinical trial navigator, but vaccines became more of a priority
**7**	(A) Engagement with community advisory board (B) Sustained relationships with community residents	(A) Recruited and maintained community advisory board (B) CHWs maintained relationships with local community and provided wrap-around services

## Data Availability

Please contact the authors for data availability.
